# Cyanobacterial NDH-1 Complexes

**DOI:** 10.3389/fmicb.2022.933160

**Published:** 2022-07-01

**Authors:** Mi Hualing

**Affiliations:** National Key Laboratory of Plant Molecular Genetics, CAS Center for Excellence in Molecular Plant Sciences, Institutes of Plant Physiology and Ecology, Shanghai, China

**Keywords:** cyanobacteria (blue-green algae), NAD(P)H dehydrogenase complex, photosynthesis, cyclic electron transport around photosystem I, CO_2_ uptake and concentrating mechanism

## Abstract

Light reaction of photosynthesis is efficiently driven by protein complexes arranged in an orderly in the thylakoid membrane. As the 5th complex, NAD(P)H dehydrogenase complex (NDH-1) is involved in cyclic electron flow around photosystem I to protect plants against environmental stresses for efficient photosynthesis. In addition, two kinds of NDH-1 complexes participate in CO_2_ uptake for CO_2_ concentration in cyanobacteria. In recent years, great progress has been made in the understanding of the assembly and the structure of NDH-1. However, the regulatory mechanism of NDH-1 in photosynthesis remains largely unknown. Therefore, understanding the regulatory mechanism of NDH-1 is of great significance to reveal the mechanism of efficient photosynthesis. In this mini-review, the author introduces current progress in the research of cyanobacterial NDH-1. Finally, the author summarizes the possible regulatory mechanism of cyanobacterial NDH-1 in photosynthesis and discusses the research prospect.

## Introduction

Cyanobacteria belong to prokaryotic photosynthetic organisms. Like other green plants, the light reaction of photosynthesis is carried out with the participation of two photosystems (photosystem I and II, PSI and PSII). Light energy absorbed by antenna pigments is transferred to the photosystem reaction centers and is converted to assimilative power (ATP and NADPH) *via* a series of electron transporters. Electron transport between PSI and PSII is mediated by the cytochrome *b6f* complex (Cyt *b6f*), coupling the translocation of protons across the thylakoid membranes. The resulting proton concentration difference (ΔpH) and membrane potential difference (Δψ) between inside and outside the thylakoid membrane drive ATP synthase (ATPase) to synthesize ATP. The electron transfer in this process is known as linear electron transport (LET). In addition, the donation of electrons from the PSI reduction side to the electron carriers between two photosystems to form circle electron transport is known as cyclic electron transport around PSI (CET-PSI). CET-PSI is also coupled with the formation of ATP, but there is no accumulation of NADPH or other reducing substances. NADPH and ATP produced by the light reaction are used for CO_2_ fixation (carbon assimilation). Compared with carbon assimilation, the light energy utilization efficiency in the light reaction is higher. The light reaction is driven by protein complexes arranged orderly on the thylakoid membrane. The protein complexes involved in LET include PSI, PSII, cytochrome *b6/f* complex, and ATPase, while the complexes involved in CET-PSI include thylakoid membrane-bound NAD(P)H dehydrogenase-like complex (chloroplast NDH or cyanobacterial NDH-1). Studies have demonstrated that NDH/NDH-1 mediated CET-PSI (Mi et al., 1992, 1994, 1995) can protect plants from photoinhibition caused by various stress environmental conditions (Endo et al., 1999; Mi et al., 2001; Wang et al., 2006) for efficient photosynthesis (Munekaga et al., 2004).

Cyanobacteria equip a series of environmental adaptations to efficiently fixate inorganic carbon (Ci) under CO_2_-limited conditions. All these adaptations, including the active uptake of Ci, the subsequent localized elevation of CO_2_ around the primary CO_2_-fixing enzyme, ribulose 1,5-bisphosphate carboxylase/oxygenase (Rubisco), and the partitioning of Rubisco into the carboxysome, are collectively considered as the carbon concentrating mechanism (CCM) (Badger et al., 2002; Badger and Price, 2003; Raven, 2003; Giordano et al., 2005). CCM requires the coordination of two systems, an inorganic carbon transporter system and the carboxysome containing Rubisco. To date, five inorganic carbon transporters have been found, including two Na^+^-dependent ${\text{HCO}}_{3}^{-}$ transporters (BicA and SbtA), one ATPase-dependent ${\text{HCO}}_{3}^{-}$ transporter (BCT1), and two CO_2_-uptake NDH-1 (type 1 NDH) complexes in *Synechocystis* sp. PCC 6803 (hereafter *Syne*6803) and other cyanobacterial strains (Ogawa and Kaplan, 2003; Ogawa and Mi, 2007; Price, 2011). Therefore, cyanobacterial NDH-1 complexes function not only in light reactions but also in carbon assimilation as an important regulator. This mini-review summarizes the recent research progress of characterization and functions of cyanobacterial NDH-1 in photosynthesis and prospects for the future research direction.

## Composition and Structural Characteristics of Ndh-1 Complex in Cyanobacteria

Through the genome comparison of chloroplast and cyanobacteria, 15 highly homologous *ndh* genes, namely *NdhA*-*O*, were found in cyanobacteria (Kaneko et al., 1996) and the corresponding homologous proteins (NdhA-O) were identified (Friedrich and Scheide, 2000; Prommeenate et al., 2004; Rumeau et al., 2005). An additional 4 proteins NdhP, NdhQ, NdhS, and NdhV were successively identified (Battchikova et al., 2011a; Nowaczyk et al., 2011; Chen et al., 2016). In contrast to the crystal structure of Complex I (Baradaran et al., 2013), cyanobacterial NDH-1 is speculated to possess an oxygenic photosynthesis-specific (OPS) domain (Birungi et al., 2010) comprised of NdhL-NdhQ, -NdhS, -NdhV. NdhL is necessary for the docking of hydrophilic subcomplex to hydrophobic subcomplex in both cyanobacteria (Battchikova et al., 2005) and plants (Shimizu et al., 2008). Deletion of NdhL lowered CET-PSI activity in cyanobacteria (Mi et al., 1992). By studying NDH-1 mutants and protein-protein interaction of multiple subunits, our group found that NdhM locates in the core of the NDH-1 hydrophilic arm of cyanobacteria and plays a key role in the assembly and activity of the NDH-1 hydrophilic arm (He et al., 2016). NdhN also affects the assembly and activity of NDH-1 (He and Mi, 2016). In contrast, although the little contribution of NdhO to CET-PSI activity, it plays an important role in respiratory metabolism under the condition of limited inorganic carbon (He and Mi, 2016). NdhV is a subunit which functions in regulation of NDH-1 activity. Mutation of *NdhV* resulted in the instability and decrease of NDH activity in Arabidopsis (Fan et al., 2015), loss of the upregulation of NDH-1 level and activity induced at high light (Chen et al., 2016), and causing heat sensitivity (Gao et al., 2016) in cyanobacteria. *E. coli* NDH-1 complex is the mode of complex I with the smallest components of protein subunits, composed of 14 subunits from NuoA to NuoN. This smallest module carries out the most basic energy conversion reaction (Friedrich and Scheide, 2000). Comparing the NDH composition of *E. coli*, cyanobacteria and chloroplasts indicate that NdhA-K is a conserved component of NDH-1 in prokaryotes and eukaryotes. However, the homologous proteins of three subunits NuoE, NuoF, and NuoG involved in NADH oxidation in *E. coli* were not found in chloroplasts and cyanobacteria. Since these three subunits contain NADH-binding sites, cofactor FMN, and iron-sulfur clusters, whether thylakoid membrane NDH-1 can directly oxidize NADH, or NADPH remains to be confirmed. Experiment using thylakoid membranes has demonstrated that ferredoxin (Fd) can donate an electron to PQ *via* cyanobacterial NDH-1 (Mi et al., 1995). At present, it is believed that the electron donor of chloroplast NDH is Fd rather than NAD (P) H (Shikanai, 2014), which is based on their laboratory study that the C-terminal of CRR31 (NdhS) subunit from Arabidopsis (Yamamoto et al., 2011; Yamamoto and Shikanai, 2013) and cyanobacterium *Syne* 6803 (Battchikova et al., 2011a), contains SH3 (Src homology 3) domain-like fold, which serves as Fd docking site. In addition, *in vitro* experiments have proved that the reduction of PQ is required for binding Fd and catalytic activity (Yamamoto et al., 2011). The C-terminal of NdhS of cyanobacteria is conserved, indicating that the electron donor element of thylakoid membrane NDH is conserved (Battchikova et al., 2011b). It was also demonstrated that thermophilic cyanobacterial Fd can interact with NDH-1 probably *via* the interaction of NdhS with NdhH or NdhI by using surface plasmon resonance (SPR) (He et al., 2015). Recently, cyanobacterial NDH-1 structures provided strong evidence that Fd binds with cyanobacterial NDH-1 *via* extensive contact with NdhI and NdhH (Pan et al., 2020; Zhang et al., 2020).

Proteomic analysis of cyanobacterial NDH-1 complexes has revealed the presence of three complexes NDH-1L/-1L' (large size), NDH-1M (medium size), and NDH-1S (small size) in *Synechosystis* PCC 6803 (Herranen et al., 2004). NDH-1L/-1L' is composed of NDH-1M, NdhD1/D2, NdhF1, NdhP, and NdhQ, which are involved in respiration and CET-PSI, while NDH-MS is composed of NDH-1M and NDH-1S (including NdhD3, NdhF3, CupA, and CupS) and NDH-MS' composed of NDH-1M and NDH-1S' (including NdhD3, NdhF3, CupB, and CupS') participate in CO_2_ uptake (Ogawa and Mi, 2007). Three types of cyanobacteria NDH-1 complexes contain NDH-1M as a skeleton. The expression of NDH-1L is stable under different growth conditions; however, the NDH-1L' has not been detected on the protein level (Zhang et al., 2004). Similar to bacterial complex I, NDH-1L forms an L-shaped architecture by analysis of the complex isolated from *Thermosynechococcus elongatus* strain earlier with electron microscopy in low resolution (Arteni et al., 2006) and recently with cryo-EM in high resolution: the previous two structures contained no NdhV (Laughlin et al., 2019; Schuller et al., 2019) and the latter two structures contained NdhV and Fd (Pan et al., 2020; Zhang et al., 2020). NDH-1MS is inducible at limiting inorganic carbon conditions and has a high uptake affinity for CO_2_, which is easily dissociated into NDH-1M and NDH-1S (Herranen et al., 2004). The NDH-1MS complex has been isolated from a *Thermosynechococcus elongatus* strain in which the C terminus of NdhL has been tagged with 6-His. This complex is easily dissociated into NDH-1M and NDH-1S complexes (Zhang et al., 2005). Single-particle electron microscopy analysis of purified thylakoid membrane components indicated that NDH-1MS had a U-shaped structure (Arteni et al., 2006). CupA is responsible for the formation of a U-shape by binding at the tip of the membrane-bound arm of NDH-1MS in both *T. elongatus* and *Syne 6803* (Folea et al., 2008). The structure of NDH-1MS has also been resolved by cryo-EM (Schuller et al., 2020). NDH-1MS' is involved in the complex of constitutive CO_2_ uptake, and its key gene *CupB* is homologous with *CupA* (Madsen et al., 2002; Shibata et al., 2002). CupB protein is dependent on NdhD4 to locate on the thylakoid membrane, and a 450 kDa NDH-1MS' complex was identified by isolation and purification (Xu et al., 2008).

The plastid coding subunits of NDH in terrestrial plant chloroplast are highly homolog to NDH-1L in cyanobacteria. In addition, plant chloroplast NDH contains at least 18 nucleus-encoded subunits formed from different subcomplexes (Ifuku et al., 2011; Ueda et al., 2012). Chloroplast NDH can form a supercomplex with a PSI core *via* two minor light-harvesting complexes I (LHCI) subunits, Lhca5 and Lhca5, for the association of PSI-LHCI (Kouril et al., 2014; Otani et al., 2018). Recently, two research works have reported the structure of chloroplast PSI-NDH supercomplex that the PSI-NDH is composed of two copies of the PSI-LHCI subcomplex, one NDH complex, and two monomeric LHCI proteins, Lhca5 and Lhca6, mediate the binding of two PSI complexes to NDH (Shen et al., 2022; Su et al., 2022).

## Possible Mechanism of Ndh-1

**Figure 1:**
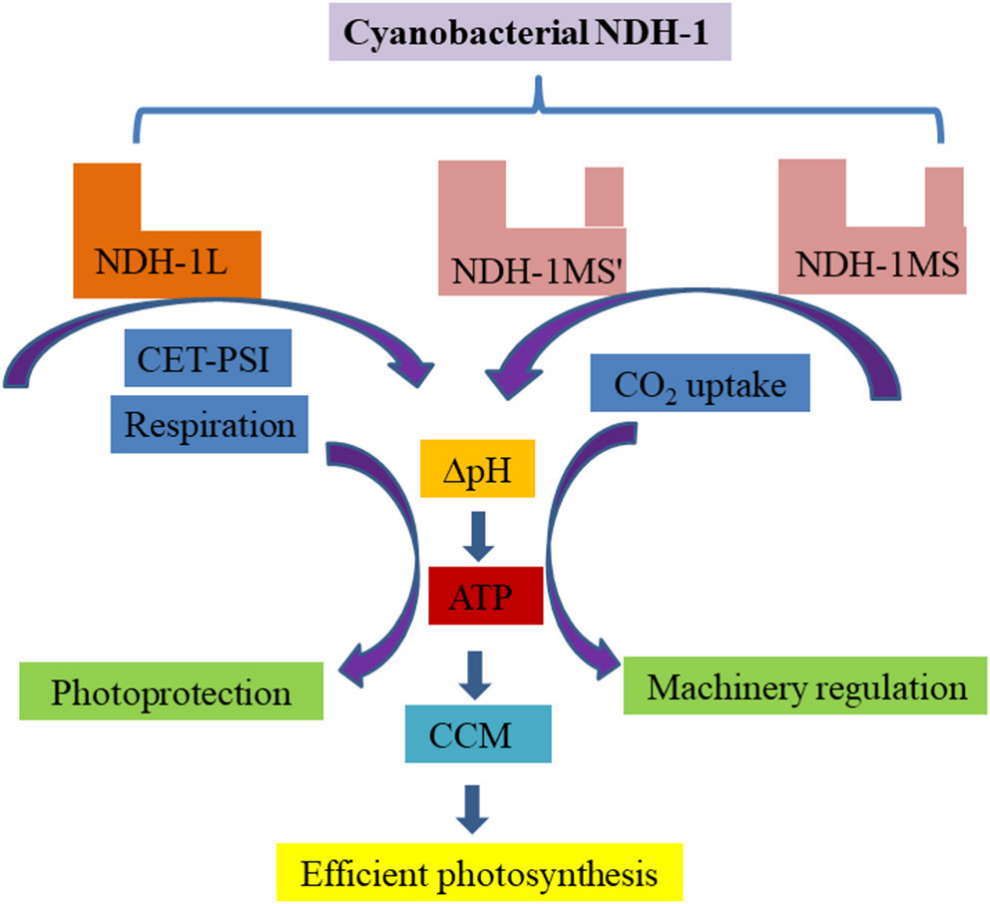
A schematic representation of the possible mechanism of cyanobacterial NDH-1 in photosynthesis. As NDH-1L is involved in CET-PSI and respiratory pathways, NDH-1MS' and NDH-MS participate in CO_2_ uptake. These pathways couple the generation of trans-thylakoid membrane proton gradient to synthesize ATP for photoprotection, photosynthetic machinery regulation, and energy supply for carbon assimilation, especially under changing environments.

As the fifth complex, chloroplast NDH only participates in CET-PSI and cyanobacterial NDH-1 is also involved in CCM, playing important role in regulating photosynthesis. Figure 1 summarizes the possible mechanism of NDH-1 based on a series of research works.

### Supplement ATP for Effective Carbon Assimilation

Cyanobacteria could survive in a low CO_2_ environment in water due to their CCMs, which can increase the CO_2_ concentration around the active site of Rubisco, the key enzyme of photosynthetic carbon assimilation, and overcome their low affinity for CO_2_, thereby effectively assimilating CO_2_ (Price et al., 1998; Kaplan and Reinhold, 1999). The CCMs and inorganic carbon transport of cyanobacteria are ATP-consuming processes. The *Syne*6803 mutant defective in NdhB (Ogawa, 1991a), NdhH, NdhJ, NdhN, or NdhM (He and Mi, 2016; He et al., 2016) completely lost CET-PSI activity mediated by HDH-1 and could not survive in the concentration of air CO_2_. The mutants lost partial NDH-1 activity and grew slower in air CO_2_ (Ogawa, 1991b; He and Mi, 2016). Klughammer et al. (Klughammer et al., 1999) used *NdhD3*-, *NdhF3*-mutants of *Synechococcus* PCC7002, to study the CO_2_ uptake efficiency. They observed that these mutants could neither induce efficient CO_2_ uptake nor efficient carbonate transport. They proposed that NDH-1 special subunits encoded by *NdhD3* and *NdhF* participate in high-affinity CO_2_ uptake. Ogawa's group proposed the involvement of NDH-1 in energy conduction and induction of a high-affinity inorganic carbon transport by studying mutants involved in CCM (Ohkawa et al., 2000). Further research indicated that deletion of NdhDs suppressed the building up of the trans-thylakoidal ΔpH resulting in the loss of the CO_2_ uptake function (Han et al., 2017). Therefore, cyanobacterial NDH-1 would provide ATP for CCM.

As the assimilation force, ATP and NADPH produced in photoreaction are used for CO_2_ assimilation. According to theoretical calculations, the ratio of ATP/NADPH required to assimilate each molecule of CO_2_ is 1.5. However, in C3 plants, due to the existence of photorespiration, the required theoretical value of ATP/NADPH increases to 1.66. When plants are in various developmental stages with different energy requirements or facing environmental changes, the fixed proportion of ATP and NADPH produced by LET often could not meet the needs of CO_2_ assimilation. Since CET-PSI only produces ATP, additional ATP can be provided (Shikanai, 2007) to meet the needs of ATP for photosynthetic carbon assimilation. In *Syne*6803, deletion of *NdhV* caused a decrease in a trans-thylakoid membrane ΔpH and loss of the upregulation of CET-PSI activity at high light (Chen et al., 2016). Also, the mutant that deleted either NdhD3 or NdhD4 significantly lowered the trans-thylakoid membrane's proton gradient (Han et al., 2017) and could not survive under CO_2_ conditions. Therefore, the ΔpH generated by NDH-1 drives the ATPase to synthesize ATP for active CO_2_ uptake and regulation of pH in the cytosol.

Hibino et al. reported that the expression of *NdhK* and *NdhI* genes was increased under high salt concentration, and CET-PSI mediated by NDH-1 was accordingly promoted (Hibino et al., 1996). Under the shock of a high concentration of salt, the *ndhB* deletion mutant could not restore its photosynthetic ability (Tanaka et al., 1997). Therefore, it is considered that CET-PSI mediated by NDH-1 is essential for cyanobacteria to adapt to salt-stressed condition. Based on plants, adapting to stressed conditions is an energy-consuming process; it is suggested that CET-PSI mediated by NDH-1 might provide extra ATP for cells to adapt to these energy-consuming reactions. The demand and proportion of ATP and NADPH in cyanobacteria and higher plants vary with the change in the photosynthetic environment or developmental stages. When the CO_2_ concentration becomes low, it is necessary to concentrate CO_2_ by consuming energy, to increase the demand for ATP. The proportion of ATP and NADPH produced by LET is certain and could not be adjusted. However, through CET-PSI, it is possible to adjust the proportion of ATP and NADPH.

### Regulation of Photosynthetic Machinery

In studying the kinetics of NADPH fluorescence (blue-green fluorescence), we observed that when the inhibitor of Calvin cycle iodoacetic acid (IAA) was added, the amplitude of rapid NADP reduction is approximately doubled in wild-type but not in the *ndhB* gene deletion mutant M55 cells. Apparently, in wild-type cells, the rate of the Calvin cycle is sufficiently high to prevent full NADP reduction even upon the onset of saturating illumination. This is possible only if there is a correspondingly high ATP supply. On the other hand, in M55 cells, full NADP reduction is obtained. This suggests that in M55 cells, ATP limits the activity of the Calvin cycle due to the lack of CET-PSI phosphorylation (Mi, 2000).

The redistribution of excitation energy between the two light systems (state transitions) is mainly to adjust the excitation imbalance of the two photosystems. It is a mechanism for plants to resist the light damage caused by excessive excitation energy. In cyanobacteria, it has been reported that NDH-1 participates in state transitions of excitation energy between PS I and II by CET-PSI. NDH-1 inactivated mutants, M55, lost the function of state transitions and were locked in state 1 (Schreiber et al., 1995). The mobility of the major accessory's light-harvesting complex phycobilisome (PBS) is related to state transitions, CET-PSI, and respiration. The immobilization of PBS at PSII inhibited the increase in CET-PSI and decrease in respiration that occurred during the movement of PBS from PSII to PSI. In contrast, the immobilization of PBS at PSI inhibited the increase in respiration and decrease in CET-PSI that occurred when PBS moved from PSI to PSII (Ma et al., 2007). State transitions in cyanobacteria, dependent on “spillover” and membrane fluidity, are induced by redox changes in the PQ pool. The light-induced state transitions induced by PBS movement caused redox poise of the PQ pool. A protein kinase known as Stt7/Stn7 has been implicated in state transitions by acting as the LHC II kinase, it seems necessary to assume that a bifurcated redox signaling pathway carries information from the PQ pool (Allen et al., 2011). NDH-I-mediated CET-PSI might participate in the redistribution of excitation energy by affecting the redox state of plastoquinone (Ma et al., 2007, 2008). However, how the redox state is sensed in cyanobacterial state transition is not clear. Plants balance their redox system by regulating CET-PSI or respiratory electron transport so that it will not be over oxidized or over-reduced. Under normal physiological conditions, when the Calvin cycle is fully active, the reduction is mainly caused by LET, and the proportion of CET-PSI is small enough to be ignored. However, when CO_2_ assimilation is decreased, NADPH and reduced ferredoxin will accumulate too much, and CET-PSI would be promoted under stressed conditions. We observed that when dark-adapted *Syne*6803 cells were moved to light, NDH-1 expression was significantly induced, and NDH-1 mediated CET-PSI and respiratory electron transport were upregulated (Mi et al., 2001). Therefore, it is suggested that NDH-1 mediated CET-PSI might co-operate with respiratory alternative oxidase transfer electrons to O_2_, thereby avoiding the over-reduction of the PQ pool under various stresses.

Our group found that the CO_2_ uptake systems NDH-1MS and NDH-1MS' are associated with a carbonic anhydrase EcaB, which catalyzes the conversion of CO_2_ into ${\text{HCO}}_{3}^{-}$ in response to high pH values, high light, or low CO_2_ concentrations, thereby helping to balance the redox state of inter photosystem electron carriers and maintain efficient photosynthesis (Sun et al., 2019).

### Photoprotection

Photosynthesis needs light energy, but the excess excitation energy would cause photoinhibition and even cause photodamage of PSII. The CET-PSI is involved in eliminating excess light energy through the mechanism of heat dissipation to protect plants from light damage caused by strong light. CET-PSI can also reduce photoinhibition by downstream regulation of PSII by generating transmembrane proton gradient (Heber, 1992). We found that the deletion of *ndhc-j-k* in tobacco leads to the accumulation of reactive oxygen species under low or high-temperature stresses (Wang et al., 2006). In addition, a low concentration of NaHSO_3_ promotes CET-PSI in *Syne*6803 (Wang et al., 2003) and tobacco under the condition of dark-light transition, alleviating photooxidative damage and improving photosynthesis by promoting photosynthetic phosphorylation mediated by NDH-1 (Wu et al., 2011, 2012).

## Conclusion Remarks

The NDH-1 plays an important role in the regulation of efficient operation of photosynthetic apparatus under changed environmental conditions. Structure bases have been made in NDH-1L and NDH-1MS. It is not clear whether there is a cross-talk between NDH-1L mediated CET-PS I and NDH-1MS/NDH-1MS' involvement in CO_2_ uptake. How does NDH-1 function in coordination with light reaction and carbon assimilation? In the future, we may use a high-resolution cryo-EM to observe and analyze active forms and states of NDH-1, and deeply study the dynamic regulation mechanism of NDH-1. With the development of molecular biology, proteomics, metabolomics, computational biology, and structural biology, it is possible to reveal the complicated regulatory network of NDH-1 in photosynthesis.

## Author Contributions

MH wrote this manuscript.

## Funding

This work was supported by funds from Synthesis Biology [2019YFA0904602] and the Strategic Priority Research Program of CAS [XDB27020106].

## Conflict of Interest

The author declares that the research was conducted in the absence of any commercial or financial relationships that could be construed as a potential conflict of interest.

## Publisher's Note

All claims expressed in this article are solely those of the authors and do not necessarily represent those of their affiliated organizations, or those of the publisher, the editors and the reviewers. Any product that may be evaluated in this article, or claim that may be made by its manufacturer, is not guaranteed or endorsed by the publisher.
